# Evaluation of Calf Muscular Function During the Recovery Phase After the Repair of an Achilles Tendon Rupture

**DOI:** 10.3389/fsurg.2019.00057

**Published:** 2019-09-24

**Authors:** Jung-Hoon Chai, Sang-Won Bae

**Affiliations:** ^1^Department of Sports Medicine, Soonchunhyang University, Asan, South Korea; ^2^Department of Orthopedic Surgery, Sunsoochon Hospital, Seoul, South Korea

**Keywords:** tensiomyography, Achilles tendon rupture repair, maximal displacement, muscle function, recovery process

## Abstract

**Background:** During the recovery phase after the repair of an Achilles tendon rupture, measuring calf muscular function is important for predicting prognosis. Tensiomyography (TMG) is a selective and non-invasive diagnostic method for skeletal muscular contractile properties based on the displacement of the muscle belly.

**Case Presentation:** Tensiomyography gives information about maximal displacement of the muscle belly (Dm), delay time, contraction time (Tc), sustain time, and relaxation time. Using Tensiomyography we evaluated a patient that had Achilles tendon rupture surgery. The contralateral normal side measurements were also performed for evaluation and comparison of the site of injury.

**Findings:** In this study, the maximal displacement of the muscle belly changed significantly compared to other parameters. The maximal displacement of the muscle belly decreased after cast removal day and increased gradually during the early recovery phase and then slightly decreased again during the late recovery phase.

**Conclusions:** These responses of the maximal displacement of muscle belly show a correlation with the recovery of muscular function.

## Introduction

The occurrence of Achilles tendon rupture happens in 2% of the population per year (1). In recent years there has been an increased interest in middle-aged and older patients in physical conditioning and joining in with athletic activities. There are two types of Achilles tendon ruptures. One is from direct trauma and another from indirect causes. Indirect causes are more frequent and result from a combination of mechanical stress and degeneration. Achilles tendon operation results are affected by several factors like age, muscle-tendon flexibility, strength, rupture site, etc. The factors that interest us most are muscle-tendon flexibility and strength of the calf muscle. TMG can evaluate the function of skeletal muscle through contraction time (Tc), maximal displacement (Dm), and other parameters.

**Figure 1:**
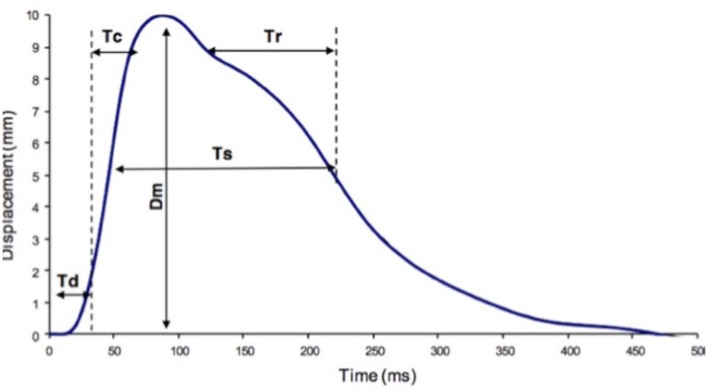
TMG parameters definition. Dm, maximal displacement; Tc, contraction time; Td, delay time; Tr, half-relaxation time; Ts, sustain time.

**Figure 2:**
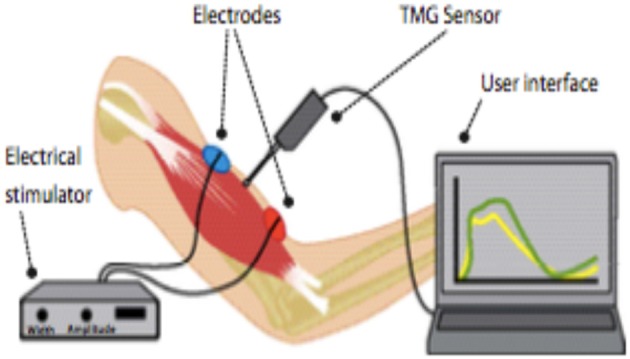
Components of TMG.

TMG was developed at the Ljublijana University in Slovenia. Early laboratory studies included evaluation of the reliability of short-term repetitive measurements and the reliability between operators, between days, and intra-correlation coefficient (2). Simunič et al. (3) found that TMG measurement indicated that the relatively simple method of TMG could be used to non-invasively estimate the %MHC-I (Myosin Heavy chain-I) in human vastus lateralis muscles. Recent studies of TMG are for pre-seasonal evaluation of football players' muscle condition and evaluation of training programs (4, 5). TMG is measured by placing 2 electrodes on each side of the muscle belly and a sensor directly on the muscle belly, electrically stimulating the calf, and then recording the muscular displacement shown in Figures 1, 2.

At this time we can identify the 5 parameters produced by TMG and explain the abbreviations.

1. Dm means the distance that is graphed by muscular contraction between the starting point and the highest point.
2. Td means the time that Dm takes to change from 0 to 10%.
3. Tc means the time that it takes to change from 0 to 90% of Dm and is related to Type I fibers of skeletal muscle.
4. Ts means the time that is taken to reach 50% of Dm.
5. Tr means the time that is taken to decrease from 90 to 50% of Dm.

This paper's purpose is to use TMG to explain calf muscular responses during recovery from Achilles tendon surgery. We evaluated the patient 4 times and compared the Dm of the calf muscle's GM (gastrocnemius medialis) and GL (gastrocnemius lateralis) throughout the 4 examinations. We are interested in using TMG with three groups. The first group is surgical and non-surgical orthopedic patients recovering from injury. The second group is athletes evaluating and maintaining their muscular function. The third group is geriatric patients with weakened muscle, especially sarcopenia.

## Methods

**Figure 3:**
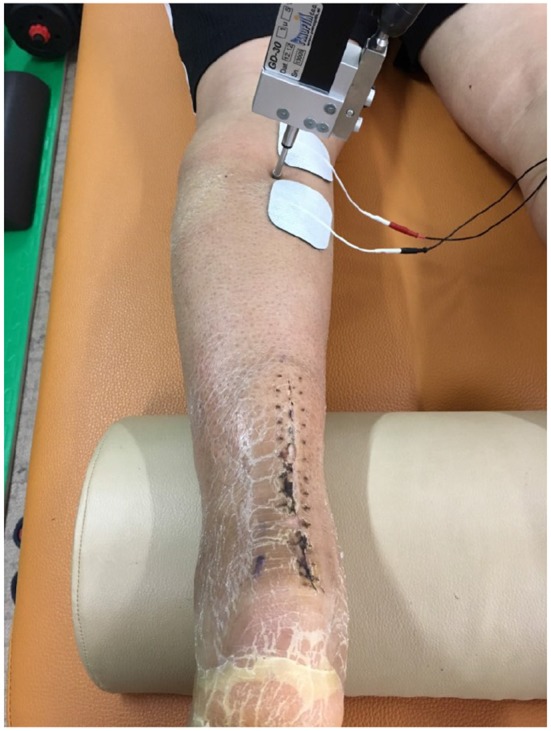
TMG measurement.

We evaluated a patient that had Achilles tendon rupture repair surgery in 2017. We explained the purpose of the study, obtained his informed consent, and received approval from Soonchunhyang University. In addition, we obtained the patient's permission of publication. The patient was a middle-aged man (range 50–55) who underwent a left Achilles tendon reconstruction on June 21, 2017, and fixed with the cast about 6 weeks. During the follow-up observation, we did the TMG test. The first measurement was on Aug. 1, 2017 (post-operative 6 weeks, cast-removal day), the 2nd on Aug. 30, 2017 (post-operative 10 weeks), the 3rd on Sept. 26, 2017 (post-operative 14 weeks), and the 4th on Jan. 1, 2018 (post-operative 31 weeks). We measured the Dm, Td, Tc, Ts, Tr of the gastrocnemius medialis, gastrocnemius lateralis, and tibialis anterior muscles. When we measured the muscle, we put the sensor directly on the muscle belly and attached 2 electrodes to each side of the sensor. Then we stimulated the muscle via the electrodes and received information through the sensor (Figure 3).

## Result

**Figure 4:**
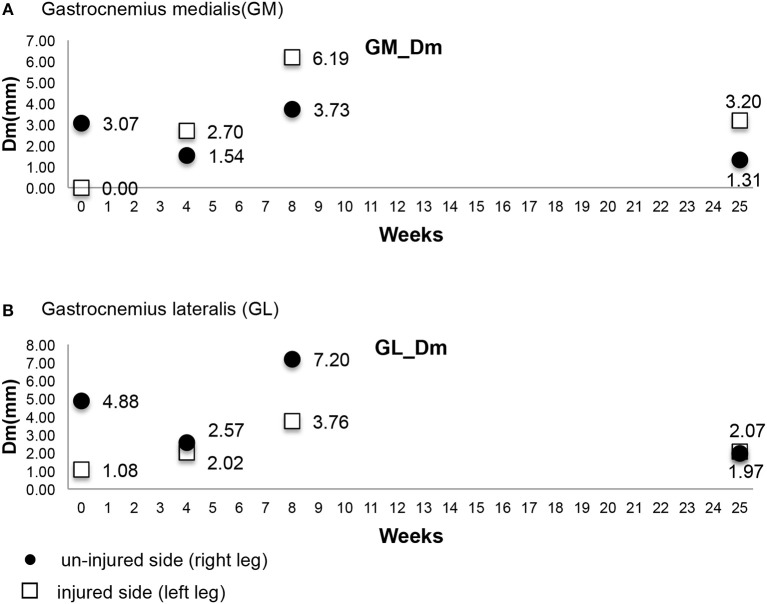
**(A,B)** Maximal displacement (Dm) of GM & GL.

In this study, we used TMG to record results of 5 parameters; the Dm, Td, Tc, Ts, Tr of the gastrocnemius medialis (GM), gastrocnemius lateralis (GL), and tibialis anterior muscles. We found clinically significant results of the Dm of GM and GL (Figure 4). The 1st date of measurement was cast-removal day and the Dm of the injured side of the GM was 0 and the Dm of the injured side of the GL was 1.08. The Dm was so low on the cast-removal day that it was nearly immeasurable. That may be caused by atrophy of the calf muscle from lack of use due to the 6 weeks in a short leg cast. The Dm of the injured side increased gradually (2nd Dm of GM was 2.70, 3rd Dm of GM was 6.19 and 2nd Dm of GL was 2.02, 3rd Dm of GL was 3.76) between the 2nd and 3rd measurement dates, but the Dm of the injured side decreased (4th Dm of GM was 3.20 and 4th Dm of GL was 2.07) on the 4th date. The 2nd measurement date was 4 weeks after cast-removal day (10 weeks post-operation). Additionally, the 3rd measurement date was 8 weeks after cast-removal day (14 weeks post-operation). The 4th measurement date was 25 weeks after cast-removal day (31 weeks post-operation). The Dm of the 4th measurement slightly decreased compared to the Dm of the 3rd the measurement. We also found that the Dm of the non-injured side of the GM and GL changed. Interestingly, the Dm of the injured side of the GM changed drastically compared to the Dm of the injured side of the GL, but the Dm of the non-injured side of the GM changed gradually compared to the Dm of the non-injured side of the GL.

## Discussion

We need a precise way of evaluating the progress and success of an Achilles tendon operation, which muscle affects the prognosis of an Achilles tendon operation, and how much the muscle function has recovered. The TMG measurement is non-invasive and can evaluate specific areas like the gastrocnemius medialis (GM) and the gastrocnemius lateralis (GL). Narici and Cerretelli (6) provided evidence that muscle atrophy induced by disuse is associated with a decrease in muscle thickness and a decrease in pennation angle.

Pišot et al. (7) found that the Dm of the vastus medialis, biceps femoris, and GM significantly increased after 35 days of bed rest. They found that the Dm and Tc are reported as more important parameters than others. Decreased Dm means there is increased stiffness of the muscle or increased muscular tone. Furthermore, increased Tc means there is a decreased muscular contractile force by muscular fatigue. Additionally, in the GM the change in Dm correlated with the decrease in muscle thickness. This finding suggested that people with hypertrophied muscles were subjected to more atrophy during unloading. Lieber (8) observed that the average physiological cross-sectional area of GM is 21.12 cm^2^ and GL is 9.72 cm^2^. Alentorn-Geli et al. (9) evaluated the neuromuscular function and later evaluated the function of the gastrocnemius muscle. We assumed that there is no affect on calf muscle contraction depending on the inflammation and dehydration of calf skin (10, 11). In this study, we found during the early recovery phase (from 1st measurement to 3rd measurement) that the Dm of the injured side increased gradually because the calf muscle slowly recovered from atrophy. However, the 4th measurement of the Dm of the injured side shows a decreased Dm compared to the 3rd measurement and did not go lower than the 2nd measurement. This means that the calf muscle of the injured side changed to a more hypertrophic state throughout the rehabilitation periods of 11 weeks. A decreased Dm means there was not only a response of muscular atrophy but also hypertrophy muscle. However, there are differential meanings of numerical value. Clinical significance of the Dm means there are changes in the pattern of the Dm. Increasing Dm means there was recovery from muscle atrophy and stiffness. The decreasing Dm means there was a change to the hypertrophied muscle. Compellingly, the Dm of the injured side of the GM changed drastically compared to the Dm of the injured side of the GL.

We also found the Dm of the non-injured side of the GM and GL changed. We can presume that the non-injured sides' Dm changed according to the muscular state of the injured side but we cannot explain this response for sure. If during the recovery phase pattern of Dm variation does not change according to the time we have to restructure the rehabilitation program or look for other causes like operation skill, duration of the cast, duration the using brace, etc.

## Conclusion

In this study's results, the Dm was determined to be significant for measuring calf muscular function post-operationally of an Achilles tendon rupture. We can assume that these changes represent conditions from disuse atrophy to a pre-injury state. Notably, the Dm of the injured side of the GM changed drastically and the non-injured side of the GM changed gradually. We can presume that the GM is more influential than the GL on the function of Achilles tendon. This study's purpose is to use TMG to evaluate the muscle affected by an Achilles tendon repair operation. This study is also the first report on this specific topic. In the future, we plan to get data from a bigger sample of patients having Achilles tendon repair operations and evaluate the effect on the calf muscle during the process of healing.

## Data Availability Statement

All datasets generated for this study are included in the manuscript and the supplementary files.

## Ethics Statement

The studies involving human participants were reviewed and approved by the ethics committee of Soonchunhyang University. The patients/participants provided their written informed consent to participate in this study. Written informed consent was obtained from the individual(s) for the publication of any potentially identifiable images or data included in this article.

## Author Contributions

J-HC and S-WB contributed conception and design of the study. S-WB organized the database. J-HC wrote the first draft of the manuscript. S-WB and J-HC wrote sections of the manuscript. All authors contributed to manuscript revision, read, and approved the submitted version.

### Conflict of Interest

The authors declare that the research was conducted in the absence of any commercial or financial relationships that could be construed as a potential conflict of interest.
